# Notable Enhancement of Phase Transition Performance and Luminous Transmittance in VO_2_ Films via Simple Method of Ar/O Plasma Post-Treatment

**DOI:** 10.3390/nano9010102

**Published:** 2019-01-16

**Authors:** Jingcheng Jin, Dongping Zhang, Xiaonan Qin, Yu Yang, Ying Huang, Huan Guan, Qicong He, Ping Fan, Weizhong Lv

**Affiliations:** 1 Shenzhen Key Laboratory of Advanced Thin Films and Applications, College of Physics and Energy, Shenzhen University, Shenzhen 518060, China; 2150100110@email.szu.edu.cn (X.Q.); 2160100104@email.szu.edu.cn (Y.Y.); 2160100101@email.szu.edu.cn (Y.H.); 2170218820@email.szu.edu.cn (H.G.); hcq13923407098@hotmail.com (Q.H.); fanping@szu.edu.cn (P.F.); 2 Key Laboratory of Optoelectronic Devices and Systems of Ministry of Education and Guangdong Province, College of Optoelectronic Engineering, Shenzhen University, Shenzhen 518060, China; 3 College of Chemistry and Environmental Engineering, Shenzhen University, Shenzhen 518060, China; lvwzh@szu.edu.cn

**Keywords:** vanadium dioxide, post-treatment, plasma irradiation, luminous transmittance, phase transition performance

## Abstract

Ar/O plasma irradiation is proposed for post-treatment of vanadium dioxide (VO_2_) films. Oxidation and surface migration were observed in the VO_2_ films following irradiation. This combined effect leads to an effective stoichiometry refinement and microstructure reconstruction in the interfacial area. A notable improvement in luminous transmittance and an enhancement in phase transition performance of the treated VO_2_ films were achieved. Compared with that of as-deposited VO_2_ films, the electrical phase transition amplitude of treated films increased more than two-fold. The relative improvement in luminous transmittance (380–780 nm) is 47.4% (from 25.1% to 37%) and the increase in solar transmittance is 66.9% (from 29.9% to 49.9%), which is comparable to or better than the previous work using anti-reflection (AR) coatings or doping methods. The interfacial boundary state proved to be crucial and Ar/O plasma irradiation offers an effective approach for further refinement of thermochromic VO_2_ films.

## 1. Introduction

Vanadium dioxide (VO_2_) undergoes a reversible metal–insulator transition (MIT) near temperatures of 68 °C [1]. During this phase transition, many of its physical properties change significantly resulting in abrupt changes in near-infrared (IR) transmittance. At high temperatures (>T_c_), the film blocks IR radiation whereas at low temperatures (<T_c_), it is allowed to pass. This unique characteristic of the phase transition is employed to regulate the solar heat flux in a manner dependent on the ambient temperature, thereby making VO_2_ a very promising material for energy-efficient, smart-window applications [2,3,4].

Much effort has been devoted to improving the thermochromic properties of VO_2_ films and limit global energy consumption in a warming climate. For the successful application of VO_2_-based energy-efficient smart windows, the requirements for high luminous transmittance and phase-transition performance must be met simultaneously. High luminous transmittance and notable IR adjustment capability are two opposing aspects in VO_2_ films. To fabricate VO_2_ films with satisfactory luminous transmittance and phase transition performance is a challenging task [5,6]. Previously, to improve its visible transmittance, much of the work was focused on coating an anti-reflection (AR) layer on a VO_2_ surface or doping another low-absorption element with VO_2_ [7,8,9,10]. These strategies were either complicated or failed to achieve the desired phase-transition performance. In VO_2_-based smart-windows research, annealing became a conventional post-treatment method that permitted remarkable modifications of the film stoichiometry as well as surface morphology [11,12]. However, in some instances, the high annealing temperatures were disastrous in fabricating toughened glass and flexible substrates [13].

In this paper, we present a simple method involving room-temperature Ar/O plasma irradiation post-treatment, which not only notable improves the visible transmittance of VO_2_ films, but also enhances its thermochromic performance. High-energy plasma irradiation is a commonly used technique to modify material surfaces. Nonetheless, Ar/O plasma irradiation for post-treatment has not been applied to thermochromic VO_2_ films. The phase-transition characteristics of VO_2_ are sensitive to the film’s stoichiometry, crystal quality, defect content, and boundary state [14,15,16,17]. The top-surface layer of the VO_2_ films bridges light transmission between film and media, and differences in boundary states of the composition and microstructure creates an anisotropy in interfacial admittance and electron mobility. Insufficient attention has been given to this interfacial state modification because controlling the interfacial configuration is difficult even during the optimized physical vapor deposition (PVD) process.

The present work aims to assess the effect of Ar/O plasma irradiation around neighboring areas of treated VO_2_ film surfaces and the corresponding impact on its MIT properties. The solo Ar and oxygen plasma have been systematically used in earlier experiments, and the better potential of property improvement was found when using the Ar/O plasma. The ratios of Ar and oxygen gas in the plasma were also systematically optimized based on earlier results. The VO_2_ films are deposited by reactive direct current (DC) magnetron sputtering and irradiated with various levels of Ar/O plasma power. We provide the results demonstrating the notable enhancement in luminous transmittance and phase-transition performance from plasma irradiation. Favorable results are achieved after properly controlling the process. Moreover, we show that electrical phase-transition amplitude increases more than two-fold while nearly preserving the thermal hysteresis width and phase-transition temperature.

## 2. Experimental Details

VO_2_ thin films with the thickness of 70 nm were deposited by DC reactive magnetron sputtering on K9 glass (D30 × 3 mm). The vacuum chamber was pumped down to a base pressure of 6 × 10^−4^ Pa, and the substrate temperature was maintained at 480 °C. The gas flow of Ar and O_2_ were 40 sccm and 2 sccm, respectively, and the working pressure was maintained around 0.5 Pa during the deposition.

The Ar/O plasma irradiation was performed in a vacuum chamber system equipped with a microwave ion source and the working frequency was 2.45 GHz (Alpha Plasma Q150). Base pressure of the vacuum system was 1.5 Pa, and the typical working temperature was 30 °C. During the plasma irradiation, the flow of Ar and O_2_ was set as 50 sccm and 100 sccm, respectively, the irradiation power range varied from 350 W to 550 W, and this lasted 30 min for each round.

The crystalline phases were identified by X-ray diffractometer (UltimaIV, Rigaku, Tokyo, Japan) with θ‒2θ coupled scanning mode (CuKα radiation with a wavelength of 0.15418 nm). The surface roughness was evaluated by Atomic force microscopy (AFM) (EasyScan2, Nanosurf, Liestal, Switzerland) with sampling range 2 μm × 2 μm. A field emission scanning electron microscope (Supra 55Sapphire, SIGMA Essential, Jena, Germany) was used to examine the surface morphology of the samples. Horiba Scientific XploRA PLUS Raman spectrum equipped with a 532 nm laser at an output power of 2 mW was used for structural properties analysis. The transmittance spectra were obtained by a spectrophotometer (Lambda 950, PE, Boston, USA) at 25 °C and 70 °C with a wavelength range of 250‒2500 nm, respectively. Temperature dependence of sheet resistances were measured by a four-probe method and the temperature range was adjusted as 25–80 °C for both heating and cooling loops.

## 3. Results and Discussion

### 3.1. Structure Characteristic

**Figure 1:**
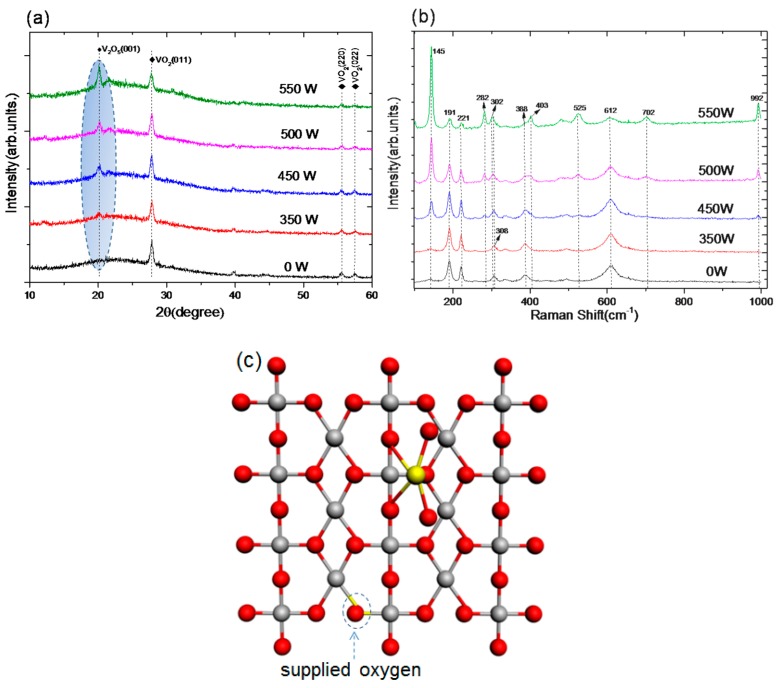
(**a**) X-ray diffraction (XRD) patterns of VO_2_ films irradiated with Ar/O plasma at different powers; (**b**) Raman spectra of Ar/O plasma irradiated VO_2_ films with different powers; (**c**) top-view of the VO_2_ cluster model showing the binding sites, the silver and red spheres represent V(+4) and oxygen atoms, and the solitary yellow sphere a V(+5) atom.

Figure 1a shows the X-ray diffraction (XRD) patterns of an as-deposited VO_2_ film and treated VO_2_ films. An intense peak located at 2θ = 27.86° and two weak peaks located at 2θ = 55.53°, 57.56° are observed in all samples. They correspond to the (011), (220), and (022) planes of VO_2_ (M) (PDF#82-0661). This indicates that the as-deposited VO_2_ layers exhibit a highly crystallized monoclinic phase with a preferential growth in the (011) lattice orientation. However, an obvious diffraction peak at 2θ = 20.13° (encircled by shaded ellipse) appears and becomes stronger as the plasma power increases. Its presence reveals that (001) V_2_O_5_ appears when the irradiation power is sufficiently high (>450 W in this instance).

The Raman spectra of VO_2_ films bombarded with Ar/O plasma (Figure 1b) show obvious Raman bands at 191, 221, 308, 388, and 612 cm^−1^, which are associated with the monoclinic phase of VO_2_ (M), all five bands corresponding to the A_g_ symmetry mode. The peaks at 308, 388, and 612 cm^−1^ correspond to the V–O vibration modes whereas the pronounced peaks of 191 and 221 cm^−1^ are assigned to the V–V vibration modes and become stronger as the power increases in the range 350–450 W. The implication is that the content of VO_2_ (M) increases from oxygen deficiency that occurs during Ar/O plasma irradiation. The new Raman peaks at 145, 282, 525, and 992 cm^−1^ belong to the V = O vibration mode in V_2_O_5_ that appears noticeably after plasma irradiation, with the increase in intensity as the bombarding power is increased [18]. The results are also in good agreement with the XRD analysis. A top-view of the model (Figure 1c) shows the binding of the clusters occurring in the VO_2_ interface area. Oxygen atoms (red spheres) may be supplied and inserted in the oxygen-vacant sites (marked by an arrow) in the VO_2_ clusters during Ar/O plasma irradiation, thereby raising the VO_2_ concentration. This may be accompanied by some additional content of V (+5) atoms (light yellow sphere) binding onto a nearby area on the surface when the Ar/O plasma irradiation power is high enough.

### 3.2. Surface Morphology

**Figure 2:**
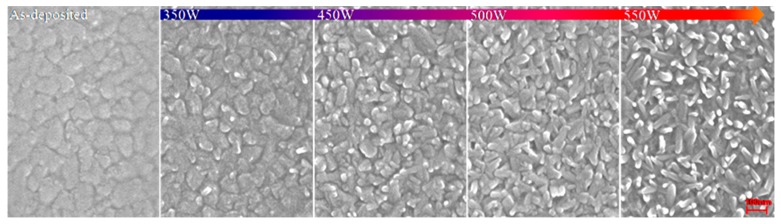
Scanning electron microscope (SEM) images of the surface morphologies inVO_2_ films irradiated with Ar/O plasma at different powers.

The surface morphologies of VO_2_ films after Ar/O plasma irradiation exhibit significant differences compared with as-deposited films. From the scanning electron microscope (SEM) images (Figure 2) flake-like nanoparticles are seen distributed evenly on the untreated VO_2_ surfaces. Small rice-like nanoparticles at first appear on the surface and grow (350 W and 450 W in Figure 2); they then gather into rod-like nanoparticles as the plasma power continues to increase up to 500 W. The rod-like shape and number of crystalline grains of VO_2_ films have significantly augmented after Ar/O plasma irradiation at 550 W.

**Figure 3:**
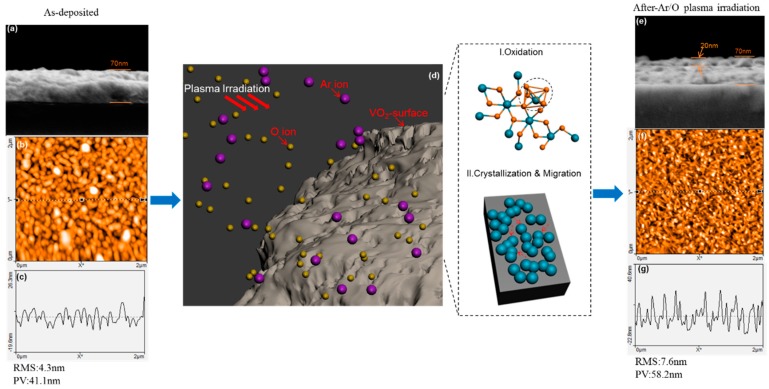
Schematic of Ar/O plasma irradiation and corresponding effect on the VO_2_ film surface (**d**); Cross-section SEM images of as-deposited VO_2_ films (**a**) and films irradiated with Ar/O plasma (500 W) (**e**); atomic force microscopy (AFM) images and line profiles of untreated VO_2_ films (**b**,**c**) and films irradiated with Ar/O plasma (500 W) (**f**,**g**).

A comparison between as-deposited VO_2_ films and films irradiated with Ar/O plasma power of 500 W of the typical morphology (Figure 3) highlights the corresponding accumulative changes on the VO_2_ surface during the irradiation process. The surface roughness is evaluated by determining the average root-mean-square (RMS) roughness and the peak–valley (PV) value obtained from atomic force microscopy (AFM) images. The underlying microstructure has undergone a conversion by the bombardment with the AFM images clearly revealing a nanoporous morphology (Figure 3b) changing into rod-like clustering (Figure 3f). Moreover, a rougher and more bumpy surface forms (Figure 3g) producing a RMS roughness and PV value of 7.6 nm and 58.2 nm, respectively. Both quantities increase sharply as the power rises because of the newly formed rod-like clusters, as evident in the SEM images (Figure 2). A SEM micrograph of a vertical section (Figure 3e) reveals that a change in boundary state results from recrystallization and surface migration in the underlying area between the top surface and the bulk of the VO_2_ film, with a penetrating depth of about 20 nm.

The irradiation of the surface causes oxidation because the energy of the co-existing Ar/O ions is high. The Ar^+^ ions colliding with the VO_2_ molecules help break the chemical bonds, and at the same time, the oxygen atoms/ions (such as O^−^, O_2_^−^) react with the V atoms to fill oxygen vacancy sites or cause further oxidation. A schematic of the interaction in a VO_2_ film during Ar/O plasma irradiation is shown in Figure 3d; a schematic enlargement (panel I) shows the crystalline structure of monoclinic VO_2_ (M) in an untreated VO_2_ film during irradiation. Either the oxygen vacancies are filled or further oxidization takes place in parts of the structure to change VO_2_ (M) into orthorhombicV_2_O_5_ (dotted circle). In addition, the Ar^+^ ion serves as an extra source of energy that may be transferred to the atomic groups (blue spheres) and induce further surface migration or crystallization near the surface area (panel II). The clusters of interfacial groups gather and reshape the surface to create new functionality. Insufficient attention has been given to this interfacial state modification because controlling the interfacial configuration is difficult during the physical vapor deposition process. Combining these two aspects, the microstructure as well as the composition in the interfacial area is reconstructed and modulated simultaneously.

### 3.3. Thermochromic Property

**Figure 4:**
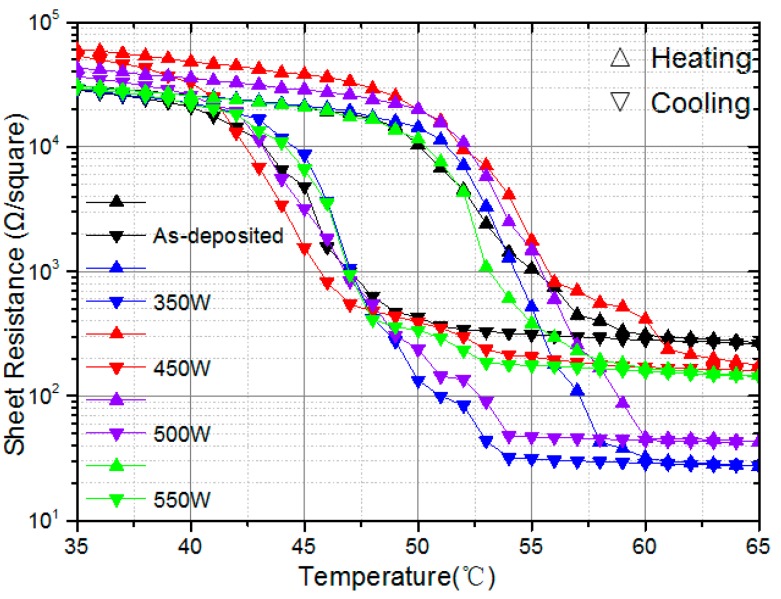
Temperature dependence of sheet-resistance in VO_2_ films treated with different Ar/O plasma powers.

**Figure 5:**
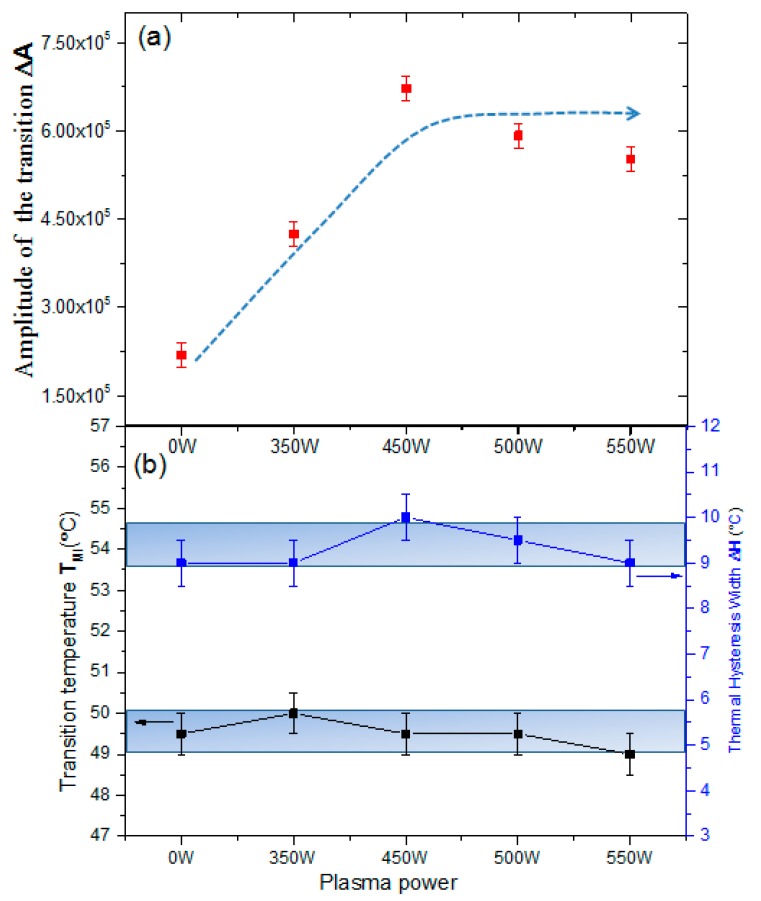
(**a**) Amplitude of transition ΔA and (**b**) phase transition temperature T_MI_ and thermal hysteresis width ΔH of VO_2_ films irradiated with Ar/O plasma of different powers.

Using the four-point probe technique, temperature-dependent sheet resistances were measured and plotted (Figure 4). The VO_2_ films exhibit semiconductor characteristics with a sheet resistance of about 10^4^–10^5^ Ω/square at room temperature, and a sharp decrease in sheet resistance is observed when increasing temperature. The ratio of the film sheet resistance before and after the phase transition determines the film’s electrical phase-transition amplitude ΔA. The average transition temperature in the heating and cooling process is defined as the film’s phase transition temperature T_MI_. The power dependence of the thermal hysteresis width ΔH and the phase transition temperature T_MI_ (Figure 5b) were obtained from standard Gaussian fitting methods by taking the derivative of the logarithmic sheet resistance with respect to the temperature of the VO_2_ films during heating and cooling cycles. Both ΔH and T_MI_ exhibit variations within the temperature ranges 9–10 °C and 53.5–54.5 °C, respectively; both were stable within the limits of error uncertainties.

From Figure 5a, ΔA of the samples obviously increases after irradiation by more than two-fold that of as-deposited VO_2_ films (from 2.19 × 10^5^ to (5–6) × 10^5^ in the range 450–550 W). In the SEM images, more boundary discontinuities between the top surface and bulk films were generated after plasma irradiation. With a reduced electron mobility, the sheet resistance of the dielectric states increases. However, the variation in composition of the VO_2_ film dominates the resistance of the metal state, altering oxygen deficiency sites or further oxidizing the VO_2_ film during plasma irradiation, as confirmed in the XRD and Raman spectra. Thus, the enhancement in the electrical phase transition amplitudes is mostly attributable to details of the stoichiometry as well as the morphology changes in the VO_2_ surface area [19].

### 3.4. Optical Performance

**Figure 6:**
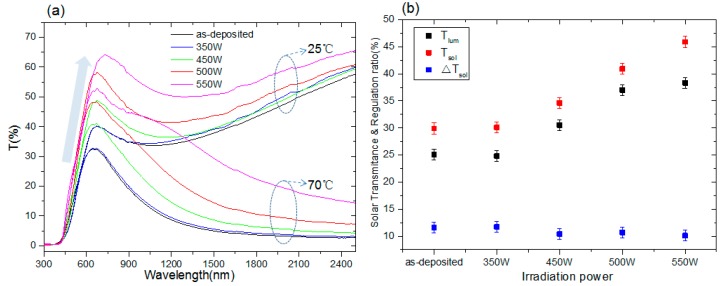
(**a**) The optical transmittance spectra of VO_2_ films bombarded with Ar/O plasma were measured at 25 °C and 70 °C using a spectrophotometer for both insulating and metallic states; (**b**) solar transmittance of T_lum_, T_sol_, and solar regulation ratio △T_sol_ in VO_2_ films after Ar/O plasma irradiation.

The optical transmittance of as-deposited VO_2_ films and treated film were measured at 25 °C and 70 °C using a spectrophotometer for both insulating and metallic states (Figure 6a). The transmittance spectra for the plasma-treated VO_2_ films in both the insulating and metallic states show sharp increases (marked by an arrow). In the insulating state, the peak transmittance in the visible range (380 nm–780 nm) increased continuously from 40% to 65%. In the infrared range, the transmittance also improved remarkably, and is only achieved with the extreme-wide-range AR coatings [20]. The transmittance of infrared solar energy and the IR-switching characteristic are calculated using the following formulae,

T_sol_ = ∫φ_sol_(λ)T(λ)d(λ)/∫φ_sol_(λ)dλ (1)

ΔT_sol_ = T_sol_ (25°C) − T_sol_ (70°C) (2)

where T(λ) is the transmittance of wavelength *λ*, φ_IR-sol_(λ) is the solar irradiation spectrum for AM1.5 in the infrared range (AM1.5 is the global standard spectrum corresponding to the Sun positioned at 37° above the horizon). The solar transmission T_vis_ is also equivalent to the standard luminous transmission T_lum_ for photopic vision in human eyes.

From the integral transmittance results (Figure 6b), T_lum_ (black square) and T_sol_ (red square) of plasma-treated VO_2_ films increase continuously from 25.1% to 38.3% and 29.9% to 45.9%, respectively, as irradiated power increases up to 550 W. The variation in the modulation ratio ΔT_sol_ (blue square) decreases slightly by about one percent at higher power. However, for VO_2_ (M) film, a calculation shows that it is difficult to achieve a sufficiently high transmittance while simultaneously preserving △T_sol_ [21]. Nevertheless, favorable results are found in the 500W group, with a relative improvement in T_lum_ (380–780 nm) to 47.4% (from 25.1% to 37%) and an increase in T_sol_ of 66.9% (from 29.9% to 49.9%) with a negligible decrease in ΔT_sol_ from 11.6% to 10.7% compared with untreated films. The high absorptance of oxygen vacancy defects can be reversed after Ar/O plasma irradiation and the rod-like clusters are reconstructed, thereby forming a new lower packing density and moth-eye like interface that may act similarly as an ‘anti-reflection coating’ [22]. Significant improvement has been realized by our simple method of Ar/O plasma post-treatment, which proved to be an efficient alternative solution compared with the former works that used AR coatings or doping methods [5,6,7,8,9,10,23,24,25].

## 4. Conclusions

In general, Ar/O plasma irradiation serves as an extra energy source that promotes oxidation and surface migration of VO_2_ molecular groups prompting micro-structure reconstruction at the VO_2_ interface. Clear evidence of enhancement in both phase transition performance and luminous transmittance has been realized by appropriately controlling the Ar/O plasma irradiation process. The long-term goal of this work is energy savings from smart-window optimization particularly suitable for building glass or plastic substrates that cannot sustain the high temperatures present in the annealing process. Opportunely, this universal method may also be extended to other situations for which low-temperature, non-destructive, effective solutions are required for further refinements of VO_2_ films.
